# Accurate Measurement in All‐inside Posterior Cruciate Ligament Reconstruction: A Technical Note

**DOI:** 10.1002/atn2.70034

**Published:** 2026-07-14

**Authors:** Daoliang Xu, Wei Yu, Zelin Jiang, Jianxia Wen, Xiaoyun Pan

**Affiliations:** ^1^ Department of Orthopaedic Surgery The Second Affiliated Hospital and Yuying Children's Hospital of Wenzhou Medical University Wenzhou Zhejiang China; ^2^ Key Laboratory of Orthopedics of Zhejiang Province Wenzhou Zhejiang China; ^3^ The Second Clinical School of Medicine Wenzhou Medical University Wenzhou Zhejiang China; ^4^ Department of Cardiology The First Affiliated Hospital of Wenzhou Medical University Wenzhou Zhejiang China

## Abstract

With the increasing rate of posterior cruciate ligament reconstruction (PCLr) and growing adoption of the all‐inside PCLr, the existing literature lacks an optimal surgical protocol for this technique. It is well recognized that variations in knee size and intraoperative positioning pose challenges in determining the key parameter—the length of the intra‐articular graft. Our technique describes an intraoperative measurement for all‐inside PCLr, specifically addressing the critical issue of imprecise graft and socket sizing. All patients achieved immediate knee stability, with negative intraoperative posterior drawer tests. This reproducible technique reduces the risk of surgical failure, and provides a practical solution for optimizing outcomes of all‐inside PCLr.

## Graphical abstract

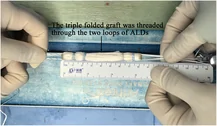

**VIDEO 1:** This surgical technique utilizes a simple self‐fabricated measuring device to accurately acquire key intraoperative parameters for all‐inside PCLr. It provides a decisive basis for intraoperative graft preparation and the creation of tibial and femoral sockets. After anesthesia, the patient is positioned supine, with the operative steps as follows: 1) Insert the arthroscope via the anteromedial/anterolateral portal to assess PCL injury and address other lesions (e.g., meniscal tears). 2) Establish posteromedial/posterolateral portals, then expose the tibial insertion of the PCL. 3) Following precise localization of the tibial and femoral insertion sites of the PCL, create 4.5 mm‐diameter thin bone tunnels in both bones, with lengths measured under direct vision. 4) Introduce a customized measuring instrument through the cortical exit of the anteromedial tibial tunnel. With the knee flexed at 70° in the anterior drawer position, measure the distance from the tibial cortical bone to the femoral tunnel entrance to determine the intra‐articular graft length. 5) Based on the intra‐articular graft length and the lengths of the bone tunnels, create appropriately sized tibial and femoral sockets. 6) Insert the graft of suitable length into the joint and maintain optimal tension. Video content can be viewed at https://doi.org/10.1002/atn2.70034.

The all‐inside surgical technique for anterior cruciate ligament reconstruction (ACLr) was first described by Morgan et al.^1^ in 1995. Subsequent refinements introduced key features such as closed‐socket tunnels, dual suspensory graft fixation, and reduced bone removal.^2^ Encouraged by promising early clinical outcomes,^3^ this technique was extended to posterior cruciate ligament reconstruction (PCLr) as early as 2013.^4^

Compared with all‐inside ACLr, the application of the all‐inside PCLr has been less frequently reported. One explanation is the substantially lower incidence of PCL injuries, which represent approximately 3% of all knee injuries, compared with more than 50% for ACL injuries.^5^ Another reason is the greater anatomical complexity of the PCL, which requires new graft preparation methods, specialized drilling, and novel fixation techniques.^6^ Despite these challenges, a 13% increase in PCLr has been reported over the past decade.^7^ The all‐inside approach addresses several limitations of conventional PCLr, particularly challenges with the “killer turn”. Clinically, reported outcomes of all‐inside PCLr have been satisfactory and are receiving increasing attention, largely due to the considerable reduction in postoperative pain.^8^ However, current all‐inside PCLr techniques lack a clearly established optimal approach. Critical factors include sockets depth and the intra‐articular distance of the PCL, which vary with intraoperative positioning and knee size. Accurate assessment of these parameters is pivotal to the success of all‐inside PCLr and must be performed with precision intraoperatively. We describe a measurement technique designed to address this challenge.

## SURGICAL TECHNIQUE

### Arthroscopic Examination and Graft Preparation

Under anesthesia, a thorough examination of the PCL is performed. The patient is positioned in a leg holder, allowing knee flexion from 0° to 120°. The PCL lesion is reassessed using routine arthroscopy; any associated injuries are treated as indicated. After evaluation, the scope is withdrawn, and an oblique anteromedial incision is made at the level of the pes anserinus. For our all‐inside PCLr technique, we prefer using a semitendinosus tendon autograft. A graft length of ≥240 mm is required to allow tripling to 75 to 90 mm, with a diameter of ≥8 mm. Graft preparation is performed as described by Lubowitz et al.^9^ the graft is looped over an adjustable loop device (ALD; Smith & Nephew, Andover, MA, USA) at both ends (Figure 1).

**FIGURE 1:**
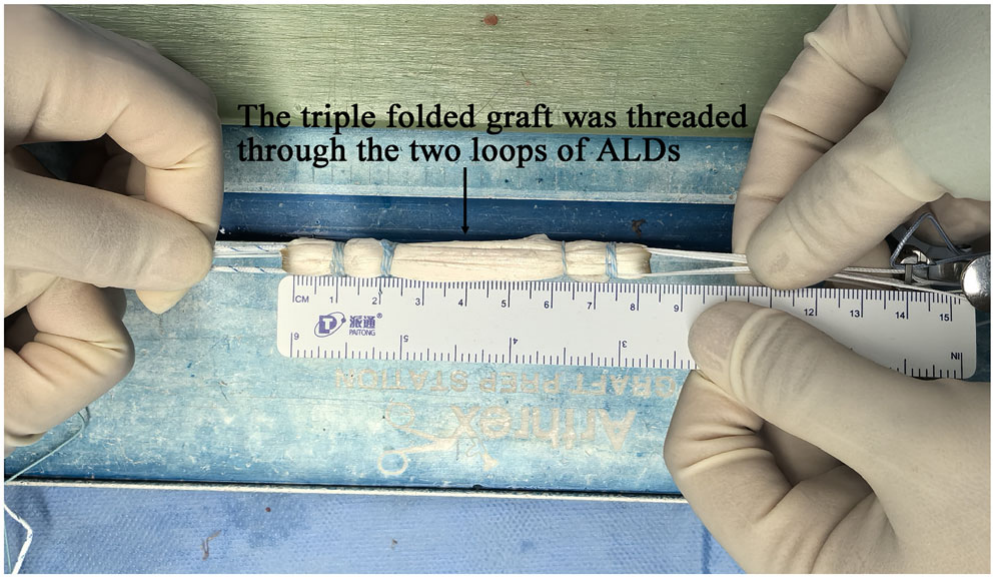
Graft preparation was performed on a dedicated graft preparation board (Graft PREP STATION, Arthrex, Naples, FL, USA). An autologous semitendinosus tendon was harvested through the anteromedial incision of the right knee. Using the technique described by Lubowitz,^8^ the graft was triple‐folded to achieve the desired length of approximately 80 mm. (ALD, adjustable loop device.)

### Accurate Measurement for PCLr Parameters

After the PCL femoral footprint has been debrided, posteromedial and posterolateral portals are established to confirm the anatomic insertion sites and ensure accurate positioning. Then, the tibial footprint is carefully cleared. Next, a PCL guide (Arthrex, Naples, FL, USA) is introduced and positioned anatomically at the tibial footprint, which is verified from the posteromedial portal. A 2.0 mm guide pin is inserted, followed by drilling of the tibial tunnel to a diameter of 4.5 mm. Subsequently, either the low‐profile reamer (Arthrex) or an inside‐out guide is positioned at the center of the femoral footprint, and a 4.5 mm tunnel is similarly created in the femur. The lengths of the tibial tunnel (defined as “a”) and the femoral 4.5 mm tunnel are measured precisely (Figure 2). At this stage, the custom measuring device is prepared (Figure 3). It is inserted into the tibial tunnel using a suture‐grasping instrument and advanced through the femoral tunnel. The total distance of the device at the femoral footprint (defined as “b”) is then recorded under direct visualization (Figure 4), while the knee is maintained at 70° of flexion in the anterior drawer position. The length of the device protruding from the tibia is defined as “c” (Figure 5), and the distance between the 2 footprints is defined as “d”. Accordingly, intra‐articular graft length is calculated using the formula: (d = b − a − c).

**FIGURE 2:**
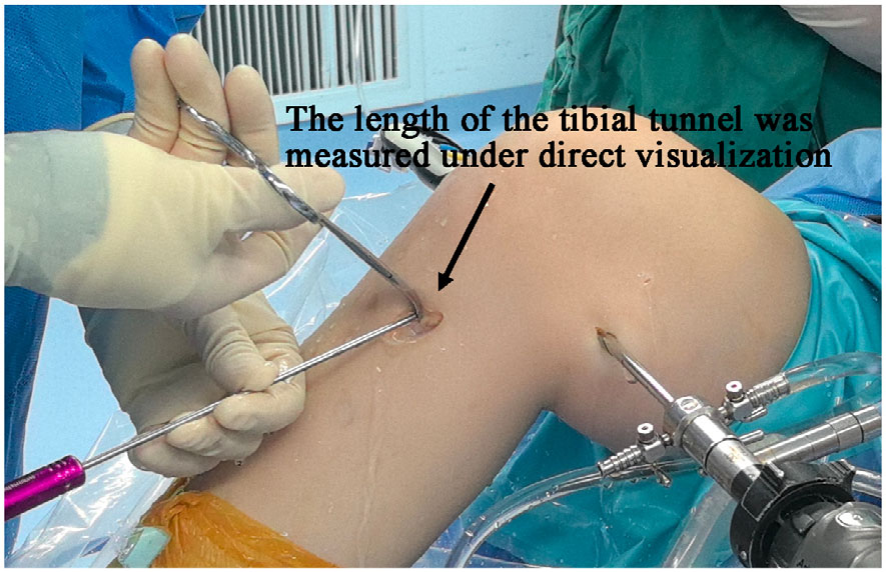
Right knee at 90° of flexion, the length of the tibial tunnel was measured with high precision under direct visualization.

**FIGURE 3:**
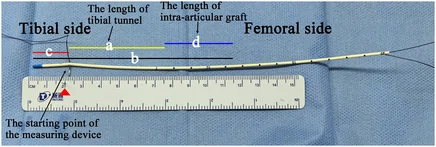
Custom‐made measuring device for determining the length of the intra‐articular graft. This device was fabricated using a graduated tube from an Anesthesia Central Venous Catheterization Kit, with a suture needle serving as the starting reference point for the tibial tunnel. Segments labeled as (a) (55 mm, the length of tibial tunnel), (b) (115 mm, the total length of the device), (c) (20 mm, the length of the device outside the tibial tunnel), and (d) (40 mm, the length of intra‐articular graft) correspond to distinct components involved in the measurement of PCL graft length. A ruler was placed in the image for scale calibration. (PCL, posterior cruciate ligament.)

**FIGURE 4:**
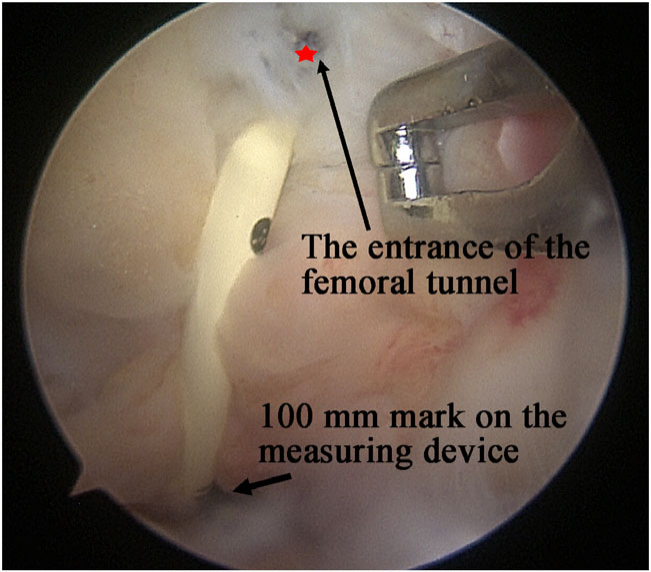
Right knee is maintained at 70° of flexion in the anterior drawer position, viewing from anterolateral portal. Measurement and recording of the distance from the anteromedial cortex of the tibia to the femoral footprint under direct visualization. The black arrow indicates the 100 mm mark on the measuring device, and the red star denotes the entrance of the femoral tunnel, which is aligned with the 115 mm position on the device.

**FIGURE 5:**
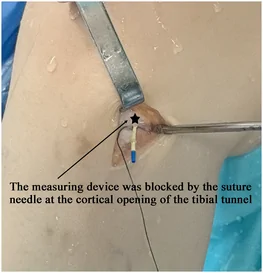
Right knee at 70° of flexion in the anterior drawer position. Intraoperative view illustrates the cortical opening of the tibial tunnel (marked with a black pentagram). The length of the measuring device that is blocked by the suture needle outside the tunnel was 20 mm.

### Accurate Creation of Bone Sockets

Based on the measured intra‐articular graft length, the appropriate lengths of the femoral and tibial sockets are planned; each socket is no shorter than 20 mm. The guiding principle is that the combined length of the tibial socket, femoral socket, and intra‐articular graft segment must exceed the total graft length, preventing graft bottoming within the sockets.^10^ The tibial socket is then created as planned, using a second‐generation retrograde drill (FlipCutter) with clockwise drilling and retrograde pressure. The femoral socket is created using the conventional technique or via an outside‐in approach with the FlipCutter, similar to its use in the tibia. Passing sutures for the tibial and femoral sockets are subsequently retrieved through the posteromedial portal and inspected to ensure the absence of soft tissue interposition or entanglement. Finally, the graft is marked at the tibial and femoral ends according to predetermined socket lengths.

### Achievement of Appropriate Graft Tension

The graft construct is first introduced into the tibial socket, with arthroscopic visualization from the posterolateral portal. The tibial ALD is advanced until it exits the anteromedial tibial cortex, after which the tibial end of the graft is secured within the socket (Figure 6A). Next, the femoral end of the graft is drawn into its socket until the femoral ALD is flipped. Under direct arthroscopic visualization, additional femoral tensioning is applied until the graft is seated to the premarked depth (Figure 6B). With appropriate tension maintained, the knee is cycled to precondition the graft. Subsequently, with the knee positioned at 70° of flexion in the anterior drawer position, the tibial ALD is deployed to achieve final graft tensioning (Figure 6C). The intra‐articular graft is then probed, and adequate reduction of ACL laxity is confirmed (Figure 7). Intraoperative physical examination showed a negative posterior drawer test (Video 1). To further improve the safety and reproducibility of the aforementioned all‐inside PCLr steps—especially for surgeons with limited experience in this technique—we summarized the core operational tips and common intraoperative risks for 5 critical procedures based on our surgical practice. These considerations directly affect graft fixation stability, measurement accuracy, and postoperative knee kinematics, and are detailed in Table 1.

**FIGURE 6:**
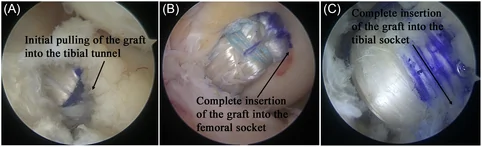
Intraoperative arthroscopic views depicting PCL graft placement and tensioning. (A) Right knee at 70° of flexion, viewing from posterolateral portal. Initial pulling of the graft into the tibial tunnel, and incomplete entrance of the marking line into the tibial tunnel is observed (arrow). (B) Complete insertion of the graft into the tunnel under the action of the femoral ALD is observed (arrow) via anterolateral portal. (C) In the anterior drawer position, final achievement of appropriate graft tension via the tibial ALD (arrow). (ALD, adjustable loop device; PCL, posterior cruciate ligament.)

**FIGURE 7:**
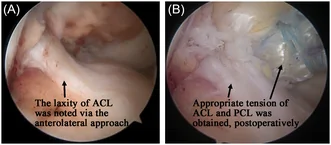
Laxity assessment of the ACL and PCL. (A) Preoperative anterolateral view of right knee showing ACL laxity (arrow) before all‐inside PCLr. (B) Postoperative anterolateral view showing the restoration of appropriate tension in both the ACL and PCL (arrow). (ACL, anterior cruciate ligament; PCL, posterior cruciate ligament.)

**TABLE 1:** Pearls and Pitfalls of This Technique

Step	Pearls	Pitfalls
Tibial socket preparation	The tibial footprint requires adequate exposure to ensure that the retrograde drill abuts the cortical bone	Soft tissue obstruction contributes to tibial socket shortening
Measurement of the intra‐articular length	Measurements shall be conducted in the 4.5‐mm bony tunnels of the tibia and femur	Eccentric displacement of the measurement device within the sockets may lead to shortening of the intra‐articular graft
Tensioning the tibial ALD	The tension of the graft and ACL should be continuously monitored under arthroscopy during tensioning	Excessive tension of the tibial ALD may result in its impingement into the tibial cancellous bone and induce knee subluxation

ALD, adjustable loop device; ACL, anterior cruciate ligament.

In cases where autograft tissue is insufficient in length or thickness for the all‐inside technique, augmentation with allograft or suture tape may serve as a viable alternative.

## DISCUSSION

Conventional PCLr has posed substantial challenges, particularly due to the relatively low bone density at the tibial insertion site. Tibial interference screws may sink into the cancellous bone, compromising fixation reliability.^11^ In contrast, the all‐inside PCLr technique has been described as safe, reproducible, and technically straightforward. This method allows the creation of 2 half tunnels with cortical suspensory fixation, enabling controlled tensioning from both sides and facilitating optimal graft tension. The bone‐preserving nature of this technique is particularly advantageous in the context of multiligament injuries.^12^

When performing all‐inside PCLr, precise intraoperative measurement of socket lengths and the intra‐articular graft segment is essential to ensure appropriate graft tension and restoration of PCL stability. However, reported data on all‐inside PCLr parameters considerably vary across studies. Femoral socket lengths are 15‐35 mm and tibial socket lengths are 20‐45 mm; Slullitel^13^ reported lengths up to 70 mm. Graft diameters are 8‐12 mm, and graft lengths are 60‐50 mm. These discrepancies are likely attributable to interindividual variations in knee size, as well as differences in surgical techniques and study methodologies. The most critical parameter—the distance between tibial and femoral tunnels, which represents intra‐articular graft length—remains uncertain, and no reliable method for its measurement has been reported. To prevent graft bottoming within the sockets, surgeons often create sockets longer than necessary, which may lead to the so‐called “bungee effect”. Excessively long sockets may also predispose the knee to subluxation if the tibial ALD is overtightened. Conversely, undersized sockets risk failure to restore appropriate graft tension. At present, most surgeons use the anatomic PCL length—reportedly 30‐38 mm—as a reference for the intra‐articular graft distance.^14^, ^15^ However, the true intra‐articular distance must be determined intraoperatively and individualized according to tunnel placement and knee size. In our technique, this critical measurement can be obtained easily and accurately using a simple device.

No standard has been established for the ideal socket length. Animal studies suggest that ≥15 mm of graft within a dense bone tunnel is required to ensure adequate tendon‐bone healing.^16^ Our measurements of the intra‐articular grafts showed a size range of 35‐45 mm. Based on this principle, we prefer a socket length of ≥20 mm on both the femoral and tibial sides, requiring a folded autograft length of ≥75 mm. When using a longer (≥90 mm) folded allograft, greater socket depth—particularly in the tibia—may be necessary. To ensure secure fixation of the ALD, it is essential to avoid full cortical perforation and to maintain ≥7 mm of thin cortical tunnel.^17^, ^18^, ^19^ In patients with osteoporosis, socket length should be decreased appropriately. For further stability, we typically create the tibial socket 5 mm longer to minimize graft slack.

In summary, our technique is easily reproducible and effective, with a demonstrable reduction in the risk of surgical failure; however, long‐term follow‐up remains necessary. The advantages and limitations of our technique are presented in Table 2.

**TABLE 2:** Advantages and Limitations of This Technique

**Advantages**		
Fabrication of the measuring device is straightforward , and the procedure is technically accessible		
Socket‐associated minimal bone trauma minimizes tunnel convergence risk in multiligament knee injuries		
Ensuring appropriate graft diameter, precise intra‐articular length optimizes graft utilization, particularly autologous grafts		
Precise measurement enhances sockets utility and mitigates the “bungee effect”		
Less bone trauma with accurate sockets length decreases postoperative pain		

**Disadvantages**		
The measurement tool requires further optimization		
The technique needs to be validated biomechanically and clinically		
Extra operative time is required for the measurement		

## DISCLOSURES

The authors (D.X., X.P.) declare the following financial interests/personal relationships which may be considered as potential competing interests: D.X. reports was provided by The Second Affiliated Hospital and Yuying Children's Hospital of Wenzhou Medical University. X.P. reports article publishing charges and writing assistance were provided by The Second Affiliated Hospital and Yuying Children's Hospital of Wenzhou Medical University. The other authors (W.Y., Z.J., J.W.) declare that they have no known competing financial interests or personal relationships that could have appeared to influence the work reported in this paper.
